# Data for DHK effect on thrombus weight, blood coagulation, blood cell counts and whole blood viscosity in deep vein thrombosis rats

**DOI:** 10.1016/j.dib.2019.104410

**Published:** 2019-08-19

**Authors:** Han Liu, Peng Li, Jin Liu, Ziqi Lu, Ping Tang, Zhaojia Li, Baoqin Lin

**Affiliations:** School of Pharmaceutical Sciences, Guangzhou University of Chinese Medicine, Guangzhou, Guangdong, 510006, China

**Keywords:** Danhong Huayu Koufuye, Thrombus weight, Blood coagulation indexes, Blood cell counts, Whole blood viscosity

## Abstract

The data presented herein are related to the research article entitled “Danhong Huayu Koufuye prevents venous thrombosis through antiinflammation via Sirtuin 1/NF-κB signaling pathway” [1]. This article describes the effect of Danhong Huayu Koufuye (DHK) on thrombus weight and blood coagulation indexes at the early and late stages of inferior vena cava stenosis-induced deep vein thrombosis in rats. In addition, the effect of DHK on blood cell counts and whole blood viscosity at the early stage were presented. The field dataset is made publicly available to enable critical or extended analyses.

**Table:** Specifications Table

Subject area	Biology
More specific subject area	Cardiology and Cardiovascular Medicine
Type of data	Table and figure.
How data was acquired	Blood coagulation indexes were measured with a coagulometer (CA-7000, Sysmex, China). Blood cell counts were detected with an automatic blood cell analyzer (XT-2000i, Sysmex, Japan). Whole blood viscosity was measured by a cone-plate viscometer (LBY-N6B, Precil, China).
Data format	Raw and analysed.
Experimental factors	Inferior vena cava stenosis-induced deep vein thrombosis rat model was established. Blood samples for blood coagulation indexes and whole blood viscosity detections need to be anticoagulated with 3.2% sodium citrate. Blood samples for blood cell counts need to be anticoagulated with 15% EDTA-K_2_.
Experimental features	Thrombus weight was measured. Blood coagulation indexes, blood cell counts and whole blood viscosity were determined.
Data source location	School of Pharmaceutical Sciences, Guangzhou University of Chinese Medicine, Guangzhou, Guangdong, 510006, China.
Data accessibility	The data are available with this article.
Related research article	H. Liu, P. Li, J. Lin, W.P. Chen, H.B. Guo, J.Y. Lin et al., Danhong Huayu Koufuye prevents venous thrombosis through antiinflammation via Sirtuin 1/NF-κB signaling pathway. J Ethnopharmacol. 2019, 241:111975. [1]

**Table:** 

**Value of the data** • The presented data indicate the antithrombotic effect of DHK at the early and late stages. • Valuable for researchers interested in the effect of traditional Chinese medicines on regulating blood coagulation, blood cell counts and blood thickness. • These data provide new insights about the mechanism of antithrombotic effect of traditional Chinese medicines.

## 1 Data

**Fig. 1:**
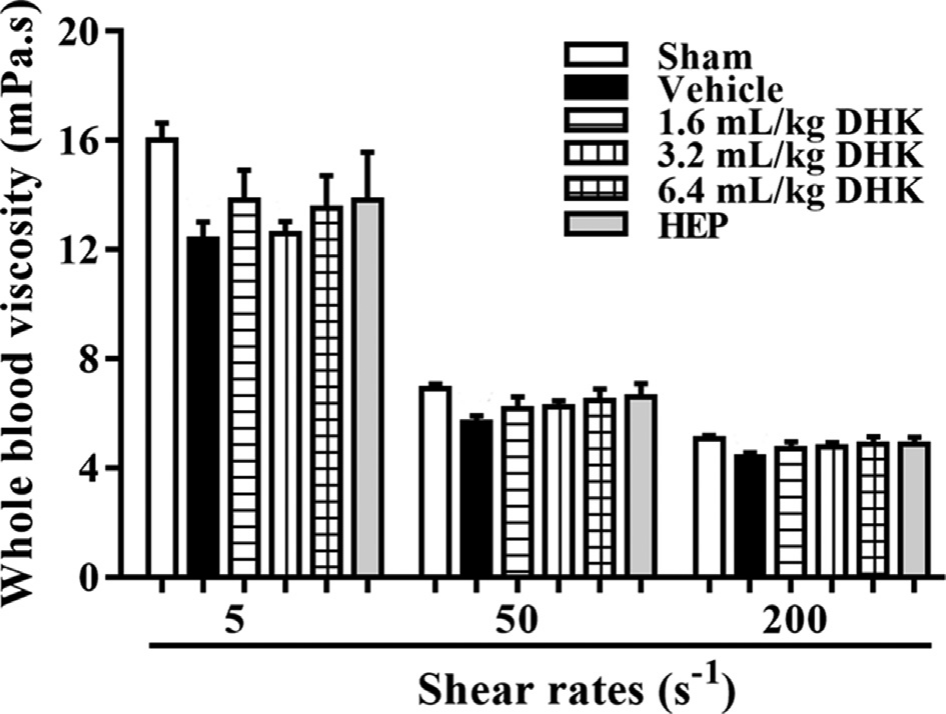
Effect of DHK administered with Method B on whole blood viscosity in IVC-stenosed rats. Data were expressed as mean ± SEM, *n* = 8.

The present data focus on the antithrombotic effect of Danhong Huayu Koufuye (DHK) in inferior vena cava (IVC) stenosis-induced deep vein thrombosis (DVT) rats. Table 1 and Table 2 showed the effect of DHK on thrombus weight and blood coagulation indexes with different administration methods, respectively. Table 3 and Fig. 1 presented the effect of DHK on blood cell counts and whole blood viscosity with method B, respectively.

**Table 1:** Effect of DHK on thrombus weight after IVC stenosis in rats (mean ± SEM, *n* = 8).

Groups		Thrombus weight (mg)
Method A	Sham	16.50 ± 0.81
	Model	15.81 ± 0.52
	DHK	15.09 ± 0.92
Method B	Sham	15.84 ± 0.75
	Model	170.00 ± 6.51
	DHK	106.26 ± 10.68
Method C	Sham	16.53 ± 0.77
	Model	200.00 ± 18.23
	DHK	125.35 ± 5.83
Method D	Sham	16.95 ± 0.70
	Model	125.00 ± 6.05
	DHK	70.01 ± 11.27

**Table 2:** Effect of DHK on blood coagulation indexes after IVC stenosis in rats (mean ± SEM, *n* = 8).

Groups		PT (s)	APTT (s)	TT (s)	FIB (g/L)
Method A	Sham	8.35 ± 0.22	18.48 ± 0.63	43.86 ± 1.33	1.97 ± 0.07
	Model	8.58 ± 0.15	15.96 ± 0.41	41.15 ± 0.71	1.94 ± 0.07
	DHK	9.11 ± 0.12	16.86 ± 0.35	47.26 ± 1.36	2.16 ± 0.09
Method B	Sham	10.02 ± 0.60	22.90 ± 1.39	42.44 ± 2.19	4.10 ± 0.18
	Model	8.70 ± 0.10	19.43 ± 1.72	43.18 ± 0.50	4.18 ± 0.20
	DHK	9.30 ± 0.47	19.63 ± 2.73	40.84 ± 0.56	4.58 ± 0.19
Method C	Sham	9.78 ± 0.22	18.45 ± 0.9	42.91 ± 1.38	2.34 ± 0.09
	Model	10.66 ± 0.14	20.00 ± 0.46	40.36 ± 1.89	2.72 ± 0.10
	DHK	11.24 ± 0.17	21.33 ± 1.43	41.01 ± 1.89	2.09 ± 0.11
Method D	Sham	ND	ND	ND	ND
	Model	ND	ND	ND	ND
	DHK	ND	ND	ND	ND

PT, prothrombin time; APTT, activated partial thromboplastin time; TT, thrombin time; FIB fibrinogen; ND means not detected.

**Table 3:** Effect of DHK administered with Method B on blood cell counts in IVC-stenosed in rats (mean ± SEM, *n* = 8).

Groups	RBC ( × 10^12^/L)	WBC ( × 10^12^/L)	PLT ( × 10^9^/L)
Sham	7.23 ± 0.10	4.32 ± 0.34	1022.20 ± 41.65
Vehicle	6.39 ± 0.11	5.49 ± 0.74	883.58 ± 39.18
1.6 mL/kg DHK	5.94 ± 0.31	4.94 ± 0.70	831.75 ± 92.08
3.2 mL/kg DHK	6.69 ± 0.16	5.31 ± 0.54	805.13 ± 64.78
6.4 mL/kg DHK	6.64 ± 0.24	4.62 ± 0.55	970.50 ± 56.94
HEP	6.38 ± 0.18	6.76 ± 0.68	983.50 ± 33.16

RBC, Red blood cells; WBC, white blood cells; PLT, platelets.

## 2 Experimental design, materials, and methods

### 2.1 Chemical and reagents

DHK was provided by Guangzhou Baiyun Mountain and Hutchison Whampo with batch number J17A002. Pentobarbital sodium was bought from Merck. Ceftriaxone sodium was purchased from Lijian. Heparin (HEP) was obtained from Hepatunn.

### 2.2 Animals

Male and female Sprague-Dawley rats (250–300 g) were provided by the Experimental Animal Center, Guangzhou University of Chinese Medicine (approved No. SYXK 2018-0001). All rats had free access to a standard diet and drinking water, and were housed in a room at 24.0 ± 0.5 °C and with a 12 h/12 h cyclic lighting schedule. All animals were cared for in compliance with institutional guidelines of Guangzhou University of Chinese Medicine. All experiments were approved by the Animal Ethics Committee of Guangzhou University of Chinese Medicine.

### 2.3 IVC stenosis-induced DVT rat model

IVC thrombus was induced as described previously [2]. In brief, rats were anesthetized with intraperitoneal injection of 36 mg/kg pentobarbital sodium and then a 2-cm incision was performed along the abdominal midline. The IVC just caudal to the left renal vein was stenosed by tying a 5-0 silk suture tightly around the vein together with an acupuncture pin (0.35 mm × 50 mm) and then removing the pin. After surgery, ceftriaxone sodium was sprinkled on the wound to prevent bacterial infection. The rats in sham group received the same surgical procedure without IVC-stenosis.

### 2.4 Experimental designation

**Fig. 2:**
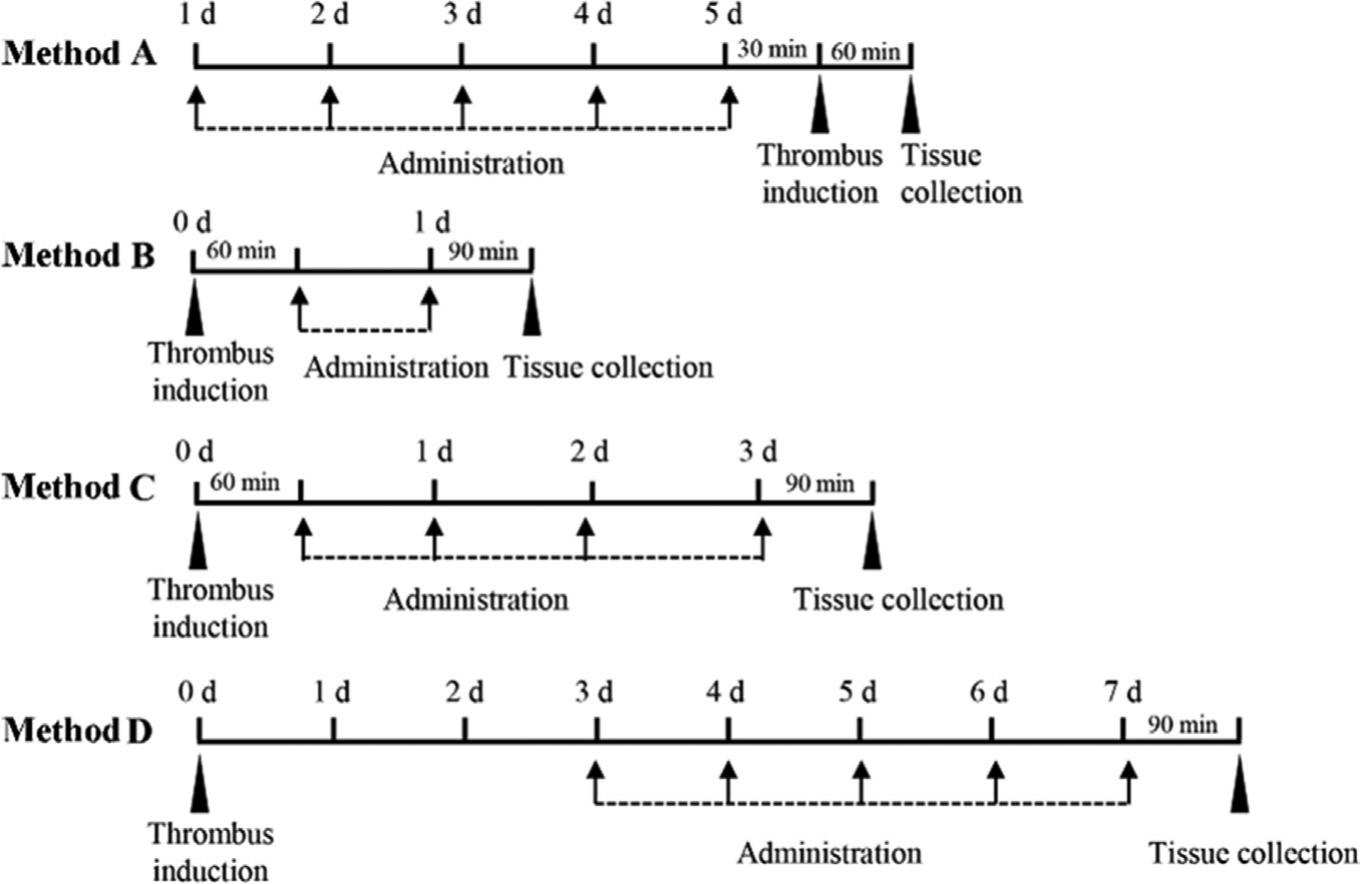
Schematic diagram of different administration methods of DHK in rats.

Different administration methods of DHK were showed in Fig. 2. Rats for analyses of thrombus weight and blood coagulation were randomly divided into three groups in each method: sham group (distilled water, p.o.), model group (distilled water, p.o.) and DHK group (3.20 mL/kg, p.o.). Rats for analyses of blood cell counts and whole blood viscosity were randomly divided into six groups using Method B: sham group (distilled water, p.o.), model group (distilled water, p.o.) and three dosages of DHK groups (1.6, 3.2 and 6.4 mL/kg/d, p.o.), and HEP group (200 U/kg/d, i.v.).

(1) prophylactic administration (Method A), rats were administered for five consecutive days. Thirty minutes after the final administration, thrombus was induced. IVCs were then collected 60 min after thrombus induction
(2) therapeutic administration, rats were administered 60 min after operation for two (Method B) or four (Method C) consecutive days, or were administered 3 days after operation for five consecutive days (Method D). IVCs were harvested 90 min after the final administration.

### 2.5 Thrombus weight

Rats were anesthetized with pentobarbital sodium. Thrombi were dissected and weighed.

### 2.6 Evaluation of blood coagulation

Blood samples from carotid artery were collected into vacuum blood collection tubes with 3.2% sodium citrate (citrate/blood: 1/9, v/v). Blood was centrifuged at 2,000×*g* for 10 min at 4 °C to obtain plasma for detections of prothrombin time (PT), activated partial thromboplastin time (APTT), thrombin time (TT) and fibrinogen (FIB) with a coagulometer (CA-7000, Sysmex, China).

### 2.7 Analysis of blood cell counts

Blood samples collected from carotid artery were collected into vacuum blood collection tubes with 15% EDTA-K_2_. Red blood cells (RBC), white blood cells (WBC) and platelets (PLT) were detected with an automatic blood cell analyzer (XT-2000i, Sysmex, Japan).

### 2.8 Analysis of whole blood viscosity

Blood samples collected from carotid artery were collected into vacuum blood collection tubes with citrate solution. Whole blood viscosity was measured by a cone-plate viscometer (LBY-N6B, Precil, China) at different share rates (5, 50 and 200 s^−1^).

### 2.9 Statistical analysis

Data were expressed as mean ± SEM (standard error of mean). Statistical significance was calculated by one-way analysis of variance test (SPSS 20.0; SPSS, Inc., Chicago, IL, USA) followed by Least-Significant Difference test. *P* < 0.05 was accepted as a statistical significance.
