# Supplementary material for: Data for DHK effect on thrombus weight, blood coagulation, blood cell counts and whole blood viscosity in deep vein thrombosis rats
Source: Data Brief. 2019 Aug 19;26:104410. doi: 10.1016/j.dib.2019.104410 (PMC6742910; doi:10.1016/j.dib.2019.104410)
Supplement: Supplementary file 2 [file mmc2.pdf]

DHK 对狭窄法诱导 SD 大鼠下腔静脉血栓（60 min）的作用

取样记录表

日期：2017.7.4

分组及编号		性别	栓重（mg）
Sham (60 min)	A1	♂	18.8
	A4	♂	13.3
	A9	♂	18.7
	A12	♂	15.5
	A18	♀	17.4
	A16	♀	18.9
	A21	♀	13.6
	A20	♀	15.8
Model (60 min)	B2	♂	13.8
	B5	♂	15.3
	B8	♂	18.3
	B10	♂	16
	B24	♀	17.3
	B13	♀	16.2
	B14	♀	15.2
	B17	♀	14.4
DHK (60 min)	C3	♂	18.3
	C7	♂	13.4
	C6	♂	15.3
	C11	♂	17.6
	C22	♀	12.3
	C15	♀	18.1
	C19	♀	12.3
	C23	♀	13.4

操作人：刘强 陆紫琪、唐[illegible]

记录人：刘强

DHK 对狭窄法诱导 SD 大鼠下腔静脉血栓（1 d）的作用

取样记录表

日期：2017.7.10

分组及编号		性别	栓重 (mg)
Sham (1 d)	A1	♂	15.5
	A5	♂	17.8
	A6	♂	18.9
	A9	♂	13.6
	A16	♀	16.8
	A18	♀	17.5
	A19	♀	14.9
	A22	♀	12.7
Model (1 d)	B4	♂	180.3
	B7	♂	170.9
	B11	♂	190.3
	B12	♂	138.4
	B13	♀	165.5
	B17	♀	186.2
	B21	♀	180.1
	B24	♀	148.3
DHK (1 d)	C2	♂	84.6
	C3	♂	103.7
	C8	♂	129.0
	C10	♂	83.1
	C14	♀	156.6
	C15	♀	103.7
	C20	♀	126.7
	C23	♀	62.7

操作人：刘[illegible]、陆[illegible]琪、唐[illegible]

记录人：刘[illegible]

DHK 对狭窄法诱导 SD 大鼠下腔静脉血栓（3 d）的作用

取样记录表

日期: 2017.7.16

分组及编号		性别	栓重 (mg)
Sham (3 d)	A2	♂	18.8
	A3	♂	15.5
	A6	♂	17.5
	A9	♂	18.9
	A13	♀	13.5
	A17	♀	15.8
	A20	♀	18.4
	A21	♀	13.8
Model (3 d)	B1	♂	157.5
	B5	♂	227.3
	B7	♂	261.3
	B10	♂	172.5
	B16	♀	158.8
	B18	♀	132
	B19	♀	221.7
	B22	♀	268.9
DHK (3 d)	C4	♂	128.9
	C8	♂	146.5
	C11	♂	104.7
	C12	♂	128.5
	C14	♀	118.2
	C15	♀	100.1
	C23	♀	138.7
	C24	♀	137.2

操作人：陆紫琪 [illegible] [illegible]

记录人：[illegible]

DHK 对狭窄法诱导 SD 大鼠下腔静脉血栓（7 d）的作用

取样记录表

日期：2017.7.20

分组及编号		性别	栓重（mg）
Sham (7 d)	A2	♂	19.0
	A5	♂	18.6
	A6	♂	15.8
	A12	♂	17.4
	A13	♀	18.7
	A16	♀	13.2
	A18	♀	15.6
	A21	♀	17.3
Model (7 d)	B4	♂	115.6
	B9	♂	142.1
	B10	♂	138.5
	B11	♂	149.6
	B14	♀	129.7
	B15	♀	110.8
	B20	♀	108.0
	B23	♀	105.7
DHK (7 d)	C1	♂	32.5
	C3	♂	71.5
	C7	♂	50.3
	C8	♂	104.4
	C17	♀	126.3
	C19	♀	47.8
	C22	♀	77.7
	C24	♀	49.6

操作人：刘涵 陆紫琪、李[illegible]

记录人：刘涵
